# Acute effects of caffeine and glucose intake on retinal vessel calibres in healthy volunteers

**DOI:** 10.1007/s10792-022-02417-z

**Published:** 2022-07-25

**Authors:** Thomas J. Gin, Noha Ali, Sivashanth Gnanasekaran, Lauren A. B. Hodgson, Lyndell L. Lim, Sukhpal S. Sandhu, Sanjeewa S. Wickremasinghe

**Affiliations:** 1 Centre for Eye Research Australia, Royal Victorian Eye and Ear Hospital, Level 1, 32 Gisborne Street, East Melbourne, VIC 3002 Australia; 2 Department of Ophthalmology, Faculty of Medicine, Assiut University, Assiut, Egypt

**Keywords:** Optical imaging, Retinal vessels, Caffeine, Glucose, Retinal artery, Retinal vein

## Abstract

**Purpose:**

To evaluate the acute effects of caffeine and glucose intake on retinal vascular calibre of healthy adults.

**Methods:**

This prospective crossover study was conducted at the Centre for Eye Research Australia (Melbourne, Australia). Standardized doses of 300 mg caffeine (approximately 3 cups coffee), 30 g glucose or 300 ml of water, were each given to 19 healthy subjects on separate days. Retinal photographs and blood pressure measurements were taken at baseline, 30-, 60- and 120-min after ingestion of each solution. Central retinal artery and vein equivalents (CRAE, CRVE) and the arterio-venule ratio were measured using computer-assisted software. The mean retinal vascular calibre measurements were compared between pre- and post-ingestion images.

**Results:**

After caffeine intake, significant reductions were observed in mean CRAE of − 9.3 μm, − 10.4 μm and − 8.5 μm and CRVE of − 16.9 μm, − 18.7 μm and − 16.1 μm at 30-, 60- and 120-min after intake when compared with baseline (*p* ≤ 0.002 for all; paired *t* test). No significant changes were observed in mean retinal vascular calibre measurements after intake of either glucose or water when compared to baseline (*p* ≥ 0.072 for all). When controlling for baseline characteristics and blood pressure measurements, only caffeine intake had a significant effect on reducing both CRAE and CRVE at all time points post ingestion (*p* ≤ 0.003 for all, multiple linear regression model).

**Conclusion:**

Caffeine is associated with an acute vasoconstrictive effect on retinal arterioles and venules in healthy subjects. Factors other than blood pressure-induced autoregulation play a significant role in caffeine-associated retinal vasoconstriction.

**Supplementary Information:**

The online version contains supplementary material available at 10.1007/s10792-022-02417-z.

## Introduction

The advent of modern digital image systems provides us the unique opportunity to reliably and objectively assess the retinal microvasculature in large population cohorts. As retinal vessels are easily imaged non-invasively in vivo, significant efforts have been made to investigate whether retinal vascular calibre measurements may reflect the state of the body’s microcirculation and, therefore, be a biomarker for cardiovascular and cerebrovascular disease. Various epidemiologic and clinical-based studies have since demonstrated that retinal vascular calibres are associated with systemic disease including hypertension [1], diabetes mellitus [2], coronary artery disease [3], stroke [4] and metabolic syndrome [5]. Nevertheless, despite advances in this field, external factors that acutely influence retinal vessel diameters are incompletely understood, nor controlled for, in many population-based studies analysing retinal vascular parameters [4, 6, 7].

Caffeine and glucose, two widely consumed constituents, have both been purported to alter ocular vessel haemodynamics. Caffeine, a natural purine alkaloid and adenosine receptor antagonist, has been shown to decrease blood flow in retrobulbar blood vessels [8] and to the optic nerve head [9] and macula [10] after oral administration in vivo. Increasing plasma glucose has been shown to exert a vasoactive effect in the eye, increasing ocular blood flow in various animal and human studies [11–13]. Similarly, chronically elevated blood glucose levels [14] and impaired fasting glucose [15] have been associated with retinal vasodilation. Any effect these popular, vasoactive substances might have on retinal vascular parameters would have significant implications on the preparation of participants for any future study analysing retinal vascular morphology. The purpose of this study is to evaluate the acute effects of standard doses of caffeine and glucose intake on the retinal vessel calibre measurements in healthy adults.

## Methods

This prospective crossover study was performed at the Centre for Eye Research Australia and was conducted in accordance with the Declaration of Helsinki of 1975. Ethics approval for this study was obtained from the Human Research Ethics Committee of the Royal Victorian Eye and Ear Hospital.

Nineteen healthy medical students and staff at the Centre for Eye Research Australia were recruited to participate in this study. Inclusion criteria for all participants were to be aged 18 years old or older with no history of systemic disease or current medication use. Potential subjects were excluded if they had diabetes, hypertension, stroke, kidney or heart disease, any known ocular diseases (including glaucoma, age-related macular degeneration) or any media opacity. Refractive error was permitted as long as this was within ± 2 diopters of emmetropia.

Three test solutions were each given in turn to the subjects, using a pattern of multiple crossover design, on separate visits over a period of up to two weeks. The order of administration of each test solution was randomly assigned. At each visit, the participant received one test solution containing a standardized dose of either (a) 300 mg caffeine dissolved in 300 ml of water, (b) 30 g sugar dissolved in 300 ml of water or (c) 300 ml of water. The caffeine dose was chosen to mirror the average intake of caffeine per day for a 70-kg adult, which in most Western countries is estimated to be 200 to 300 mg per day [16].

To minimize diurnal variation effects, testing was performed in the morning at each visit for each participant. All participants were instructed to fast from midnight and avoid caffeinate drinks for 12 h prior to each study visit**.** At each visit, after pupil dilation with tropicamide 0.5%, retinal photography from both eyes, was obtained at baseline before the administration of any substance, and then at 30-, 60- and 120-min post ingestion. The time interval for measurements is based on the peak plasma concentrations of caffeine occurring between 30 and 120 min after oral administration [17].

### Retinal photography and retinal vessel calibre measurements

Digital retinal photography was used to assess retinal vessel calibre and was performed according to a standardized protocol with a 45º digital nonmydriatic Canon CR6-45NM camera (Canon, Tokyo, Japan) with photographic fields taken of each eye centred on the optic disc. Right eye retinal photographs were selected for measurements. If the right eye fundus photograph was of insufficient quality for retinal vascular calibre measurement for any participant, the left eye was used.

**Fig. 1:**
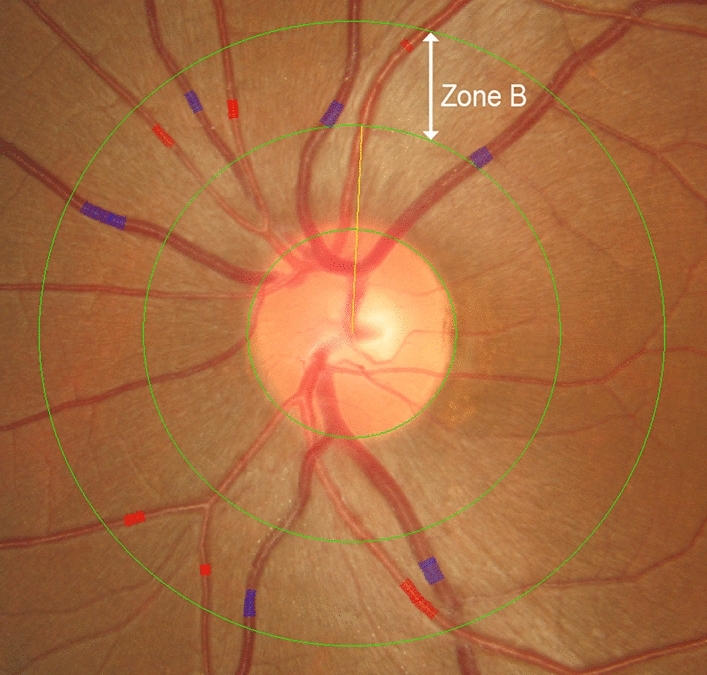
Disc-centred photograph of the left optic nerve head, highlighting the “Big 6” arterioles and venules used to calculate the central retinal artery equivalent and central retinal vein equivalent

Retinal vascular calibre was measured using standardized computer-assisted semi-automated imaging software Integrative Vessel Analysis (IVAN, University of Wisconsin, WI) [18]. For each photograph, the six largest retinal arterioles and six largest venules coursing through an area of 0.5–1.0 disc diameter from the disc margin were traced using the semi-automated computer software (Fig. 1, Zone B). Further manual corrections were made by a masked grader (N.A.) as required. Using the revised Knudtson–Parr–Hubbard formula, measured calibres of the retinal arterioles and venules were summarized into a one-sum value as the central retinal artery equivalent (CRAE) and central retinal vein equivalent (CRVE) respectively [19]. This remains the gold-standard approach for quantifying retinal vessel calibres [18, 20]. The arteriovenous ratio (AVR) was calculated as the ratio of CRAE to CRVE.

### Blood pressure measurements

Systemic blood pressure (BP) was measured at each visit using an automated device with an appropriately sized cuff with the subjects in a seated position. A single measurement was taken after at least a 5-min period of rest at each time point. In the first nine recruited subjects, BP was only measured at baseline. However, to study the correlation between the results and the change in the BP, the subsequent ten subjects had BP measured at baseline, 30-, 60- and 120-min. Mean arterial pressure (MAP) was calculated by the formula utilizing systolic blood pressure (SBP) and diastolic blood pressure (DBP): MAP = DBP + 1⁄3 (SBP–DBP)**.**

### Statistical methods

Retinal vascular calibre measurements (CRAE, CRVE, AVR) and blood pressure measurements (SBP, DBP, MAP) were assessed as continuous variables. Normality was assessed for all analyses, and appropriate non-parametric methods were applied as necessary. Comparisons of vessel calibre measurements and blood pressure measurements between baseline and post-ingestion measurements at 30-, 60- and 120-min were performed by a paired sample t-test to account for the within-eye pairing of measurements. The relationships between the retinal vascular calibres (CRAE, CRVE, AVR) and the blood pressure measurements (SBP, DBP, MAP) were calculated with the Pearson correlation coefficient. Significance levels were set at *p* < 0.05. The impact of each independent variable (including baseline characteristics, blood pressure parameters and agent ingested at baseline) on CRAE and CRVE at 30-, 60- and 120- minutes was analysed with a multivariate regression model. All statistical analyses were conducted using SPSS software (version 24.0, SPSS Inc., Chicago, USA).

## Results

Nineteen subjects (14 females, 5 males) were recruited in this study. The mean age of the participants was 29.5 ± 8.2 years (range 22–53) with an average BMI of 22.8 ± 2.6 (range 19.2–29.0)**.** The habitual caffeine intake of participants ranged from 0 and 4 cups of coffee per day (mode, mean consumption ± SD): 1, 1.5 ± 0.9 cups per day. A standard 8 oz cup of coffee will have approximately 100 mg of caffeine [16].

**Fig. 2:**
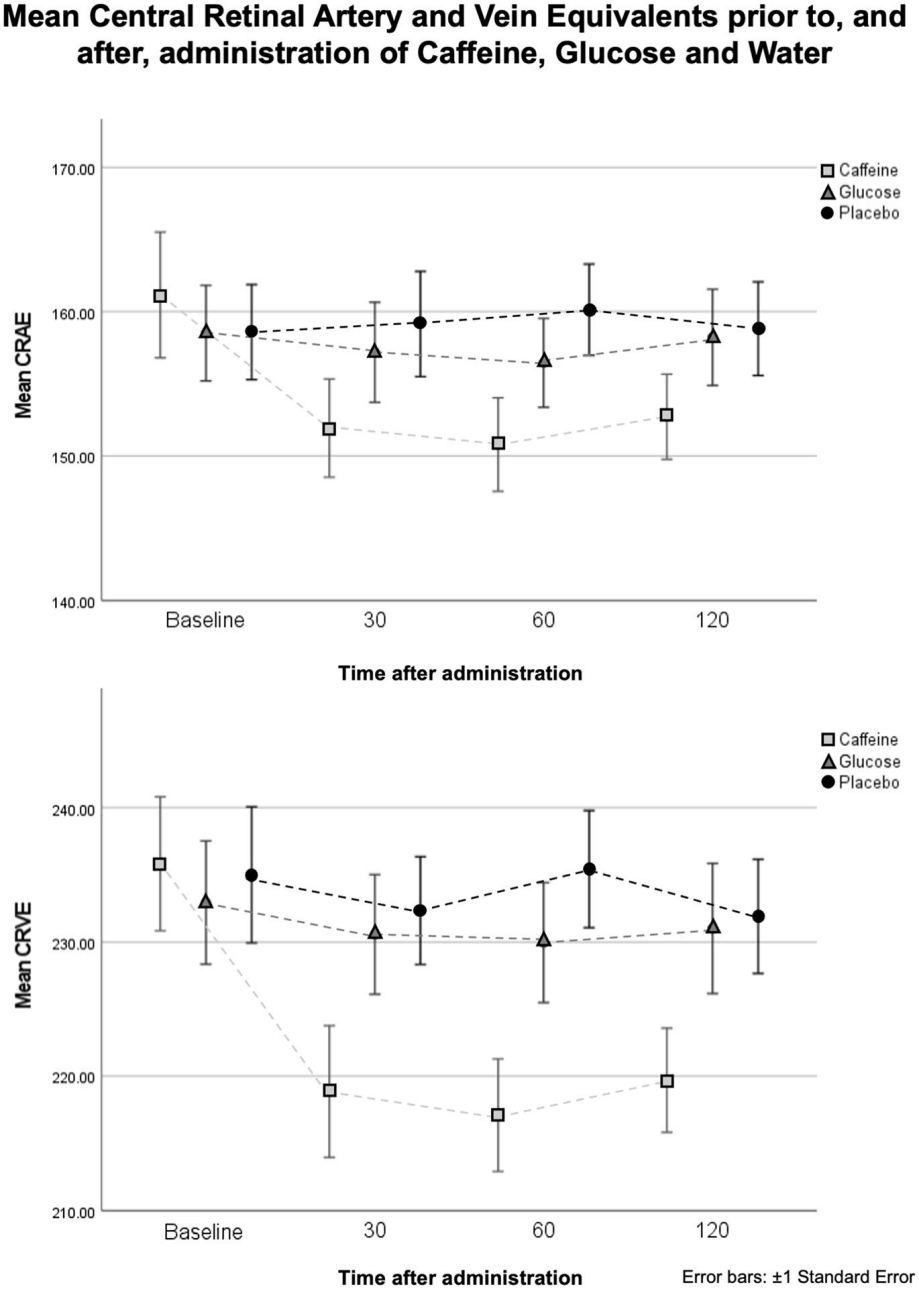
Mean central retinal artery and vein equivalents (CRAE, CRVE) prior to, and 30-, 60- and 120-min after administration of caffeine, glucose and water. Significant reductions were observed in mean CRAE and CRVE at 30-, 60- and 120-min after caffeine intake when compared with baseline (*p* ≤ 0.002 for all; paired *t* test)

Mean retinal vascular calibre measurements prior to, and at 30-, 60- and 120-min following, intake of 300 mg of caffeine, 30 g of glucose and 300 ml of water, are demonstrated in Table 1 and Fig. 2. Significant reductions were observed in mean CRAE and CRVE at 30-, 60- and 120-min after caffeine intake when compared with baseline (*p* ≤ 0.002 for all; paired *t* test). Significant increases were observed in mean AVR at 30- and 60-min after caffeine intake when compared to baseline (*p* ≤ 0.002 for both). The increase in AVR at 120-min after caffeine intake, when compared to baseline, approached statistical significance (*p* = 0.065). No significant changes were observed in mean CRAE, CRVE and AVR at 30-, 60- and 120-min after intake of either glucose or water alone when compared to baseline (*p* ≥ 0.072 for all).

**Table 1:** Retinal vascular calibre measurements prior to, and 30-, 60- and 120-min after administration of caffeine, glucose and water

	Baseline	30 Minutes		60 Minutes		120 Minutes	
	Mean ± SD	Mean ± SD	*p*†	Mean ± SD	*p*†	Mean ± SD	*p*†
*Caffeine*							
CRAE	161.2 ± 18.5	151.9 ± 13.6	**0.001**	150.8 ± 12.9	**0.002**	152.7 ± 12.5	**0.002**
CRVE	235.8 ± 21.1	218.9 ± 19.6	** < 0.001**	217.1 ± 16.8	** < 0.001**	219.7 ± 16.5	** < 0.001**
AVR	0.68 ± 0.04	0.70 ± 0.04	**0.036**	0.70 ± 0.05	**0.001**	0.70 ± 0.04	0.065
*Glucose*							
CRAE	158.5 ± 14.4	157.2 ± 15.1	0.392	156.5 ± 13.1	0.292	158.2 ± 14.5	0.796
CRVE	232.9 ± 20.0	230.6 ± 19.4	0.171	230.0 ± 19.0	0.517	231.0 ± 21.1	0.294
AVR	0.68 ± 0.04	0.68 ± 0.04	0.922	0.68 ± 0.04	0.859	0.68 ± 0.04	0.605
*Water*							
CRAE	158.6 ± 14.3	159.1 ± 15.9	0.592	160.1 ± 13.8	0.072	158.8 ± 14.2	0.832
CRVE	235.0 ± 22.1	232.3 ± 17.5	0.187	235.4 ± 19.0	0.840	231.9 ± 18.5	0.168
AVR	0.68 ± 0.04	0.68 ± 0.04	0.179	0.68 ± 0.04	0.383	0.69 ± 0.04	0.086

*CRAE* Central retinal artery equivalent; *CRVE* Central retinal vein equivalent; *AVR* Artery to vein ratio

^†^Paired sample *t*-test comparison with baseline data

The significant *p* values (*p* < 0.05) given in bold

Overall, there were no significant changes in mean SBP, DBP or MAP from baseline to 30-, 60- and 120- minutes (*p* > 0.21). The results of the mean blood pressure measurements prior to, and at 30-, 60- and 120-min following, intake of caffeine, glucose and water alone are available in Online Resource 1. Significant elevations however were observed in mean SBP at 30-, 60- and 120-min after caffeine intake when compared with baseline (p ≤ 0.039 for all; paired t test), while DBP and MAP values did not change following caffeine ingestion throughout the study (p ≥ 0.156 for all). Significant reductions were observed in mean DBP and MAP at 120-min after glucose intake when compared to baseline (p ≤ 0.017 for both). Otherwise, no other significant changes in SBP, DBP or MAP were observed at all time points after intake of either glucose or water alone when compared to baseline (p ≥ 0.168 for all). No significant correlations were found between blood pressure markers (SBP, DBP, MAP) and retinal vascular calibre measurements at the baseline, 30-, 60-, and 120-min after the intake of caffeine; CRAE (p ≥ 0.142 for all; Pearson correlation coefficient), CRVE (p ≥ 0.174 for all) and AVR (p ≥ 0.149 for all).

The results of the multivariate regression model looking at characteristics for subjects (including age, gender, baseline mean SBP, change in mean SBP from baseline and the agent ingested at baseline) and their influence on CRAE and CRVE at 30-, 60- and 120- minutes are available in Online Resource 2. After controlling for other characteristics, there was a significant effect of caffeine intake, − 13.12 μm (95% confidence interval [95% CI] − 5.08, − 21.16), *p* = 0.003 on CRAE at 30 min post ingestion. Similarly, caffeine reduced CRAE by − 7.99 μm (95% CI − 12.59, − 3.39), *p* = 0.002 at 60 min and by − 14.28 μm (95% CI − 21.55, − 7.01), *p* = 0.001 at 120 min. Likewise, after controlling for other characteristics, CRVE reduced by − 13.71 μm (95% CI − 5.70, 21.72), *p* = 0.002, 30 min after caffeine ingestion. At 60 min and 120 min, a reduction of − 11.89 μm (95% CI − 18.25, − 5.53), *p* = 0.001 and − 16.88 μm (95% CI − 26.53, − 7.22), *p* = 0.001 respectively, were observed.

After controlling for other characteristics, there was a significant effect of gender on CRVE of − 19.03 μm (95% CI − 36.82, − 1.25), *p* = 0.04, at 120 min post intake, but not at 30- or 60- minutes post ingestion. Similarly, mean SBP at baseline reduced CRVE by − 0.46 μm (95% CI − 0.86, − 0.06), *p* = 0.03 at 120 min, but not at 30- or 60- minutes post intake.

## Discussion

In the current study, we observed a significant narrowing of retinal arterioles and venules in healthy subjects for at least 2 h after 300-mg of oral caffeine intake. No significant change in retinal vascular calibre was seen following ingestion of glucose or water alone. An increase in systolic blood pressure was observed following caffeine intake, however after controlling for blood pressure measurements and baseline characteristics, only caffeine intake had a significant effect on reducing both central retinal artery and vein equivalents at all time points up to 120 min post ingestion.

Our study adds to the growing evidence observing the significant effect of caffeine intake on ocular blood flow and vascular resistance. Macular blood flow and ocular blood velocity have been shown to significantly reduce after oral caffeine ingestion [9, 10]. Caffeine has also been shown to accompany an increase in the resistive index of ophthalmic vasculature via colour doppler ultrasonography [8]. Our results also complement studies demonstrating the acute vasoconstrictive effect accompanying caffeine ingestion on optical coherence tomography angiography. Optical coherence tomography angiography peripapillary and macular vessel densities, as well as, markers of macular flow area have been shown to significantly decrease one hour after oral caffeine intake [21, 22].

To our knowledge, Terai and colleagues have published the only previous study to investigate the acute effect of caffeine on retinal vascular calibres [23]. They observed a significant 4.9% and 6.7% reduction in arterial and venule diameter respectively, in young healthy subjects, one hour after oral ingestion of 200-mg of caffeine [23]. Our observations of a 6.5% and 7.9% reduction in CRAE and CRVE respectively, 1 h after oral intake of 300 mg of caffeine, align with their findings. Although it is possible our larger dose of caffeine may explain the larger percentage reduction in vascular calibre accompanying caffeine, these results are not directly comparable. Our method to measure the six largest arterioles and venules, rather than a single major temporal artery and venule [23], ensure our data is the more robust representation of retinal vascular diameters.

Our findings of a mean change in CRAE and CRVE of 10.4 μm and 18.7 μm respectively, 60-min after caffeine ingestion, is comparable to the magnitude of retinal vascular calibre changes reported to be associated with a significant increased risk of cardiovascular events. As an example, a meta-analysis by McGeechan et al. [24] found that wider venules and narrower arterioles were each associated with an increased risk of coronary heart disease in women with pooled hazard ratios of 1.16 per 20-μm increase in venular calibre and 1.17 per 20-μm decrease in arteriolar calibre. Similarly retinal venular calibre has been independently associated with an increased risk of stroke events with a pooled hazard ratio of 1.15 per 20-μm increase in venular calibre [25]. Thus, the magnitude of the caffeine-associated reductions in CRAE and CRVE found in our study, could be of significance to studies evaluating the association of retinal vascular calibres with subclinical and clinical cardiovascular disease.

The mechanisms by which caffeine induces vasoconstriction are not entirely clear. Autoregulatory myogenic smooth muscle contraction in response to elevated BP has been suggested to play a key role [26]. However, our study observed no significant correlations between all blood pressure markers and retinal vascular calibre measurements, suggesting factors other than blood pressure-induced autoregulation, play a significant role in caffeine-associated vasoconstriction. Vasoconstriction may be mediated by caffeine’s role as an adenosine-receptor antagonist at physiological concentrations. Adenosine is an endogenous vasodilative molecule. Adenosine intravitreal injections has been shown to lead to retinal arteriolar dilatation in animal studies [27] and intravenous adenosine has been observed to induce ophthalmic vasodilation in healthy subjects [28]. In our present study, the decrease in CRAE and CRVE after caffeine intake was associated with an increase in AVR, indicating the associated decrease in central retinal vein diameter was greater than the corresponding decrease in the central retinal artery diameter. It is possible this represents the active response of smooth muscle cells surrounding the retinal venules reacting to an increase in perfusion pressure through sympathetic innervation [23]. Nonetheless further studies are required to elucidate the underlying mechanism of retinal vasoconstriction in response to caffeine.

In our present study, no significant change in retinal vascular calibre was observed within 2-h following ingestion of 30 g of glucose. Various animal and human studies have shown acute increases in plasma glucose is associated with an increase in ocular blood flow [11–13]. However, these animal studies suggest this increase in retinal blood flow is due to a rise in retinal blood velocity and that retinal branch arteriolar and venular diameters remain unchanged, consistent with our findings [11, 29]. In healthy subjects, retinal vascular diameters measured on video fluorescein angiography, remained unchanged with increasing plasma glucose level up to 300 mg/dl, via hyperglycemic insulin clamps, when compared to baseline [12, 30]. Our results are in keeping with the studies cited above, where a lower peak plasma glucose level would be expected in our healthy subjects following oral ingestion of 30 g of glucose.

Given the ubiquitous nature of caffeine consumption [31], knowledge of the acute physiological effects of caffeine on retinal vascular calibres is crucial if we are to accurately interpret clinical and population-based studies investigating retinal vascular calibre measurements. Our findings of caffeine-induced retinal vasoconstriction have implications for the preparatory methodology for any future clinical and population-based studies investigating retinal vascular parameters. We recommend all patients abstain from caffeine ingestion prior to retinal vascular imaging. Exclusion of this potential confounder in future studies may allow clearer delineation of associations between retinal vascular imaging assessments and clinical and subclinical cardiovascular and metabolic outcomes.

Our study has several strengths. Firstly, our crossover study design assisted to exclude non-drug related effects on our measured outcomes. Secondly, our use of standardized computer-assisted semi-automated imaging software to measure retinal vascular calibres ensured our measured parameters were a robust cross-sectional representation of retinal vessel calibre. The current study however is limited by small participant numbers. The results of our regression analyses, particularly the statistically significant effect of gender and mean SBP at baseline, on the change in CRVE at 120-min post ingestion of caffeine, may have been influenced by the small sample size. Finally, our participants may be younger than most participants enrolled in clinical studies evaluating the association between cardiovascular events and retinal vascular calibre measurements. It is possible the vasoconstrictive response associated with caffeine found in our cohort may not represent those seen in cohorts of older participants.

In conclusion, our study has demonstrated the acute vasoconstriction associated with caffeine on retinal arterioles and venules in healthy subjects. Our results indicate that future epidemiologic and clinical-based research on retinal vessel calibres should take into account the possibility of caffeine-associated changes in retinal vessel measurements.

## Supplementary Information

Below is the link to the electronic supplementary material.

Supplementary file1 (PDF 76 KB)

Supplementary file2 (PDF 65 KB)
