# Supplementary material for: Acute effects of caffeine and glucose intake on retinal vessel calibres in healthy volunteers
Source: Int Ophthalmol. 2022 Jul 25;43(1):207–14. doi: 10.1007/s10792-022-02417-z (PMC9902432; doi:10.1007/s10792-022-02417-z)
Supplement: Supplementary file 1 — Supplementary file1 (PDF 76 KB) [file 10792_2022_2417_MOESM1_ESM.pdf]

Online Resource 1. Mean blood pressure parameters prior to, and 30-, 60- and 120-minutes after administration of caffeine, glucose and water.

	Baseline	30 minutes		60 minutes		120 minutes	
	Mean ± SD	Mean ± SD	*p*†	Mean ± SD	*p*†	Mean ± SD	*p*†
**Caffeine**							
SBP	111.1 ± 13.9	120.4 ± 6.9	**0.039**	117.7 ± 15.9	**0.011**	117.7 ± 15.3	**0.032**
DBP	76.7 ± 7.1	78.7 ± 8.7	0.472	77.9 ± 10.4	0.692	77.5 ± 7.3	0.719
MAP	88.2 ± 8.8	92.6 ± 11.1	0.156	91.2 ± 11.3	0.219	90.9 ± 9.5	0.235
**Glucose**							
SBP	112.3 ± 9.7	110.6 ± 8.8	0.388	112.1 ± 9.7	0.928	111.9 ± 10.7	0.897
DBP	75.6 ± 6.7	74.0 ± 6.1	0.378	75.9 ± 6.4	0.912	70.1 ± 5.4	**0.016**
MAP	87.8 ± 6.0	86.2 ± 5.1	0.320	88.0 ± 6.2	0.947	84.0 ± 5.7	**0.017**
**Water**							
SBP	110.4 ± 15.0	109.8 ± 12.3	0.803	107.9 ± 8.0	0.467	109.6 ± 9.6	0.814
DBP	74.7 ± 6.9	75.9 ± 8.6	0.569	72.0 ± 8.3	0.182	75.9 ± 6.3	0.580
MAP	86.6 ± 9.4	87.2 ± 9.3	0.750	84.0 ± 6.7	0.168	87.1 ± 6.2	0.825
**Overall**							
SBP	111.3 ± 12.6	113.6 ± 13.6	0.209	112.6 ± 12.0	0.422	113.1 ± 12.2	0.320
DBP	75.7 ± 6.7	76.2 ± 7.9	0.672	75.3 ± 8.6	0.783	74.5 ± 7.0	0.366
MAP	87.5 ± 7.9	88.7 ± 9.0	0.386	87.7 ± 8.7	0.890	87.4 ± 7.6	0.885

SBP, systolic blood pressure; DBP, diastolic blood pressure; MAP, mean arterial pressure.
†Paired sample t-test comparison with baseline data.
The significant p values ($p < 0.05$) given in bold values.
