# Supplementary material for: Acute effects of caffeine and glucose intake on retinal vessel calibres in healthy volunteers
Source: Int Ophthalmol. 2022 Jul 25;43(1):207–14. doi: 10.1007/s10792-022-02417-z (PMC9902432; doi:10.1007/s10792-022-02417-z)
Supplement: Supplementary file 2 — Supplementary file2 (PDF 65 KB) [file 10792_2022_2417_MOESM2_ESM.pdf]

Online Resource 2. Multiple linear regression model for change in central retinal artery equivalent from baseline, adjusting for baseline factors

Characteristics	CRAE [µm] (95% CI)	*p*
**30 minutes post ingestion**		
Age (years)	-0.11 (-4.9, 0.27)	0.54
Gender (Male vs. Female)	-4.56 (-9.43, 18.55)	0.51
BMI	1.13 (-0.92, 3.19)	0.26
Mean SBP at baseline	0.14 (-0.14, 0.43)	0.31
Change in mean SBP from baseline	0.11 (-0.31, 0.52)	0.59
Ingested agent (Caffeine vs no caffeine)	-13.12 (-5.08, -21.16)	**0.003**
Model; R^2 = 0.48, F= 3.17, p= 0.02		
**60 minutes post ingestion**		
Age (years)	-0.10 (-0.29, 0.10)	0.31
Gender (Male vs. Female)	7.73 (-0.95, 16.42)	0.08
BMI	-0.03 (-1.31, 1.26)	0.97
Mean SBP at baseline	-0.01 (-0.20, 0.19)	0.94
Change in mean SBP from baseline	0.13 (-0.14, 0.39)	0.33
Ingested agent (Caffeine vs no caffeine)	-7.99 (-12.59, -3.39)	**0.002**
Model; R^2 = 0.71, F= 7.55, p< 0.001		
**120 minutes post ingestion**		
Age (years)	-0.10 (-0.36, 0.34)	0.95
Gender (Male vs. Female)	-4.90 (-18.29, 8.49)	0.46
BMI	-0.63 (-2.65, 1.30)	0.52
Mean SBP at baseline	-0.23 (-0.53, 0.07)	0.12
Change in mean SBP from baseline	-0.29 (-0.69, 0.10)	0.14
Ingested agent (Caffeine vs no caffeine)	-14.28 (-21.55, -7.01)	**0.001**
Model; R^2 = 0.47, F= 3.30, p= 0.02		

CRAE; Central Retinal Artery Equivalent (microns)
CRVE; Central Retinal Vein Equivalent (microns)
BMI; Body mass index
SBP; Systolic blood pressure (mmHg)

Multiple linear regression model for change in central retinal vein equivalent from baseline, adjusting for baseline factors

Characteristics	CRVE [µm] (95% CI)	*p*
**30 minutes post ingestion**		
Age (years)	0.44 (-0.33, 0.42)	0.81
Gender (Male vs. Female)	9.44 (-4.51, 23.39)	0.17
BMI	1.67 (-0.38, 3.72)	0.11
Mean SBP at baseline	0.27 (-0.10, 0.56)	0.06
Change in mean SBP from baseline	-0.05 (-0.46, 0.37)	0.82
Ingested agent (Caffeine vs no caffeine)	-13.71 (-5.70, 21.72)	**0.002**
Model; R^2 = 0.62, F= 5.62, p= 0.001		
**60 minutes post ingestion**		
Age (years)	-0.15 (-0.42, 0.12)	0.27
Gender (Male vs. Female)	4.95 (-7.07, 16.97)	0.40
BMI	-0.33 (-2.11, 1.45)	0.70
Mean SBP at baseline	-0.11 (-0.38, 0.17)	0.43
Change in mean SBP from baseline	0.29 (-0.08, 0.65)	0.12
Ingested agent (Caffeine vs no caffeine)	-11.89 (-18.25, -5.53)	**0.001**
Model; R^2 = 0.73, F= 8.62, p< 0.001		
**120 minutes post ingestion**		
Age (years)	0.12 (-0.35, 0.58)	0.61
Gender (Male vs. Female)	-19.03, (-36.82, -1.25)	**0.04**
BMI	-2.11 (-4.80, 0.57)	0.12
Mean SBP at baseline	-0.46 (-0.86, -0.06)	**0.03**
Change in mean SBP from baseline	-0.31 (-0.84, 0.22)	0.24
Ingested agent (Caffeine vs no caffeine)	-16.88 (-26.53, -7.22)	**0.001**
Model; R^2 = 0.49, F= 3.46, p= 0.02		

CRAE; Central Retinal Artery Equivalent (microns)
CRVE; Central Retinal Vein Equivalent (microns)
BMI; Body mass index
SBP; Systolic blood pressure (mmHg)
